# Mapping and Functional Analysis of a Maize Silkless Mutant *sk-A7110*

**DOI:** 10.3389/fpls.2018.01227

**Published:** 2018-08-21

**Authors:** Yan Zhao, Yongzhong Zhang, Lijing Wang, Xueran Wang, Wei Xu, Xianyu Gao, Baoshen Liu

**Affiliations:** ^1^State Key Laboratory of Crop Biology, College of Agronomy, Shandong Agricultural University, Tai’an, China; ^2^Agricultural Technology Promotion Center of Yanzhou, Jining, China

**Keywords:** maize, silkless, map-based cloning, RNA-seq analysis, jasmonic acid

## Abstract

The maize (*Zea mays*) stigma, which is commonly known as silk, is indispensable for reproduction and thus for grain yield. Here, we isolated a spontaneous mutant *sk-A7110*, which completely lacks silk; scanning electron microscopy showed that the *sk-A7110* pistils degenerated during late floret differentiation. Genetic analysis confirmed that this trait was controlled by a recessive nuclear gene and *sk-A7110* was mapped to a 74.13-kb region on chromosome 2 between the simple sequence repeat markers *LA714* and *L277*. Sequence analysis of candidate genes in this interval identified a single-nucleotide insertion at position 569 downstream of the transcriptional start site in *Zm00001d002970*, which encodes a UDP-glycosyltransferase; this insertion produces a frameshift and premature translational termination. RNA-sequencing analysis of young ears identified 258 differentially expressed genes (DEGs) between *sk-A7110* and the wild type (WT), including 119 up- and 139 down-regulated genes. Interestingly, most DEGs related to jasmonic acid (JA) synthesis were up-regulated in the mutant compared to WT. Consistent with this, the JA and JA-Isoleucine (JA-Ile) contents were significantly higher in *sk-A7110* ears than in WT. At the same time, RNA-sequencing analysis of tassels showed that *sk-A7110* could reduce the number of tassel branches in maize by down-regulating the expression of *UB2* and *UB3* genes. Our identification of the *sk-A7110* mutant and the responsible gene will facilitate further studies on female infertility research or maize breeding.

## Introduction

Maize (*Zea mays* L.) is a monoecious plant with separate male (tassel: formed by apical meristem) and female (ear: formed by axillary meristems) inflorescences. The inflorescence development in maize is determined by three different types of meristems: the spikelet-pair meristems (SPMs), spikelet meristems (SMs) and floral meristems (FMs). Male and female inflorescences undergo almost identical development before the initiation of sex determination. Early female and male florets have same floral organ primordia, which include one lemma, one pair of lodicules, three stamen primordia, and one pistil primordium. Mature unisexual flowers form via selective abortion (or degradation) of the floral organ primordia (Cheng et al., 1983; Calderon-Urrea and Dellaporta, 1999). In sex determination, the pistil primordium of the male tassel and the lower floret and stamen primordium of the ear degenerate, ultimately producing a functional unisexual flower. Therefore, the fate of the pistil has a central role in sex determination in maize.

Studies of mutants with alterations in sex determination have shown that pistil development is associated with complex processes such as selective cell death, cytoprotection and signal transduction (Cheng et al., 1983; Calderon-Urrea and Dellaporta, 1999; Chuck et al., 2007; Hultquist and Dorweiler, 2008). Among these processes, the regulation of phytohormone signaling plays a crucial role in fate determination of the pistil. Studies of various male mutants, which lack female organs, showed that endogenous gibberellic acids (GAs) play a major role in promoting stamen degradation and inhibiting the abortion of the pistil primordium in the male panicle (Calderon-Urrea and Dellaporta, 1999). The developing pistils can produce high levels of GA, which is conductive to the maturation of the silk and inhibit the development of stamens (Dellaporta and Calderon-Urrea, 1994; Eveland et al., 2014). Analysis of adenosine phosphate-isopentenyltransferase (IPT), a key cytokinin biosynthesis enzyme, suggested that high concentrations of cytokinin can inhibit pistil abortion in lower florets and determine pistil cell fate during sex determination (Young et al., 2004). Studies showed that brasinosteroids (BRs) plays an important role in stamen and anther development (Hartwig et al., 2012). Polar auxin transport plays an important role in meristem development and floral organ morphology. For example, maize *Barren Inflorescence 2* (*Bif2*) encodes a serine threonine protein kinase that controls auxin polarity transport through regulation of the subcellular localization of PIN proteins. Its mutant *bif2* has reduced number of female ears and significantly reduced number of tassel branch and floret numbers (McSteen et al., 2007). In male inflorescences of the *tasselseed 1* (*ts1*) and *ts2* mutants, endogenous JA contents are significantly reduced, and external JA treatment restores stamen development (Acosta et al., 2009; Chuck, 2010). These findings suggest that JA mediates pistils abortion in maize tassels, but its effect on the development of pistils in the ear requires further study.

In this study, we identified a spontaneous maize mutant *sk-A7110*, which completely lacks silk and has significantly reduced tassel branching. In *sk-A7110* mutant ears, the pistils degrade at late stage of floret differentiation. We mapped the *sk-A7110* locus and identified a single-nucleotide insertion in *Zm00001d002970*, which encodes a UDP-glycosyltransferase, leading to a frame shift and early termination. RNA-seq analysis showed that mutation of this gene affects many genes and multiple pathways. JA contents were significantly higher in mutant ears than in wild type (WT). These results suggest that *Zm00001d002970* is the responsible gene for *sk-A7110* and that it is related to JA metabolism.

## Materials and Methods

### Plant Materials and Mapping Population

Wild type maize inbred lines A7110, B73, P2, and 80044 were used in this study. The *sk-A7110* mutant was identified from the maize inbred line A7110, and *sk1*, whose phenotype is very similar to that of *sk-A7110*, was obtained from the Maize Genetics Cooperation Stock Center^1^. Since *sk-A7110* is completely masculinized, the *sk-A7110* stock was maintained by mating *sk-A7110* with *+*/*sk-A7110* siblings. One near isogenic line population was constructed by crossing *+*/*sk-A7110* with *sk-A7110*. Three BC_1_ backcross populations were produced by crossing *sk-A7110* with inbred lines B73, P2, and 80044, followed by backcrossing with *sk-A7110*. These three BC_1_ segregating populations and one near isogenic line population were used for segregation analysis of the mutant phenotype and the P2/*sk-A7110*//*sk-A7110* population was used for mapping purposes. All materials were grown at the Experimental Station of Shandong Agricultural University (Tai’an, China).

### Scanning Electron Microscopy

For scanning electron microscopy, female inflorescences and male inflorescence at different developmental stages were fixed in 2.5% glutaraldehyde solution overnight and dehydrated in an ethanol series. The samples were critical point dried, and glumes were manually dissected to reveal developing florets. The samples were sputter coated with palladium for 60 s and viewed on a Hitachi S-4700 at an accelerating voltage of 2kV. Images were processed using Adobe Photoshop CS2.

### Genetic Analysis and Molecular Mapping

The phenotype of the F_1_ plants and three BC_1_ populations was investigated. The main phenotypes we investigated were: whether vegetative growth was normal, whether there were filaments, the size of female ears, and the number of male branches.

For genetic mapping of the *sk-A7110* locus, bulked segregant analysis (BSA) and simple sequence repeat (SSR) molecular markers were used. 228 SSR markers distributed over all 10 chromosomes were used to screen polymorphism between P2 and *sk-A7110.* Two DNA pools (a mutant pool and a WT pool) prepared from 10 individuals/pool of the BC_1_ population are used to detect selected polymorphic primers. For fine mapping, new SSR markers between *umc1555* and *umc1448* were developed by SSR Hunter 1.3 and Primers 5.0. Polymorphic molecular markers used for gene mapping are in **Supplementary Table S1**.

### Transcriptome Analysis

A P2/*sk-A7110*/*sk-A7110* population was planted in the filed during the summer of 2016. Because mutant and WT plants were indistinguishable before appearing of the silks, the plants were genotyped using molecular markers linked to the *sk-A7110* gene at the seedling stage. Heterozygous normal (*sk-A7110/+*) and mutant plants (*sk-A7110/sk-A7110*) in the population were marked with closely linked markers. The young ears of WT and mutants at the nine-leaf stage were quickly transferred to a centrifuge tube and frozen in liquid nitrogen. Additionally, the young tassels of mutants (*sk-A7110/sk-A7110*) and the WT (*SK-A7110/SK-A7110*) of its near isogenic line at the nine-leaf stage were quickly transferred to a centrifuge tube and frozen in liquid nitrogen. Per biological replicate samples from 15 plants were pooled. Three WT biological replicates and three mutants once were made. Total RNA was extracted using TRIzol (Invitrogen) according to the manufacturer’s protocol and treated with RNase-free DNase I (Takara). RNA-seq libraries construction and sequencing were both performed at Novogene Bioinformatics Technology Co. Ltd. (Beijing, China). RNA-seq data of ears and tassels were deposited in the National Center for Biotechnology Information (NCBI) Sequence Read Archive (SRA) under accession number SRP155767 (BioProject ID: PRJNA483126) and SRP155763 (BioProject ID: PRJNA483310), respectively.

The reads were mapped to the maize reference genome B73 AGPv3 using TopHat. Differential expression analysis of six samples was performed using the DESeq R package, and *p*-values were adjusted to control the false discovery rate. Unigenes with an adjusted *p (q) value < 0.05* identified by DESeq were considered to be differentially expressed.

GO annotation and GO enrichment analysis (corrected *p-value < 0.05*) of DEGs were performed to further investigate their functions. GO enrichment analysis of the DEGs was conducted using GOseq R packages (Young et al., 2010) based on Wallenius non-central hyper-geometric distribution. GO terms with corrected *p (q) value < 0.05* were considered to be significantly enriched among the DEGs. To further investigate the biological functions and interactions of genes, pathway-based analysis was conducted using KEGG (Kanehisa et al., 2008).

### Endogenous Hormones Measurement

Samples were prepared as described for transcription analysis. Tissue samples were stored at -80°C prior to solvent extraction. Hormone quantification was performed via vapor phase extraction for sample preparation and gas chromatography-mass spectrometry (GC-MS) as described (Stitz et al., 2011). Hormone levels were analyzed with a Shimadzu LC/MS-8040 as described (Stitz et al., 2011).

### Allelism Analysis

Since both *sk1* and *sk-A7110* are silkless, a heterozygous plant (+/*sk-A7110*) from the offspring of *sk-A7110*/*sk-A7110* and *SK-A7110*/*SK-A7110* was used as the female parent and a *sk1* homozygous mutant (*sk1/sk1*) was used as the male parent for hybridization. The hybrid progeny were planted and their female phenotypes scored. A segregation ratio of wild type: silkless mutant plants of approximately 1:1 would indicate that *sk1* and *sk-A7110* are allelic mutants, and the presence of all normal progeny would indicate that they are not allelic.

### Quantitative RT-PCR Analysis (qRT-PCR)

To validate the DEGs identified by RNA-seq and to analyze the expression of other genes, quantitative reverse-transcription PCR (qRT-PCR) was performed. The samples were prepared as described for transcriptome analysis. Samples were pooled from 5 plants per biological replicate (three biological replicates of WT and mutant). Total RNA was isolated from frozen samples using an EASYspin Plus Plant RNA Kit (Aidlab). High-quality first-strand cDNA was generated using a HiFiScript gDNA Removal cDNA Synthesis Kit (Calbiotech). The maize *ACTIN* gene was used as an internal control. Gene-specific primers used for qRT-PCR are provided in **Supplementary Table S3**.

## Results

### Phenotypic Characterization of *sk-A7110*

**FIGURE 1:**
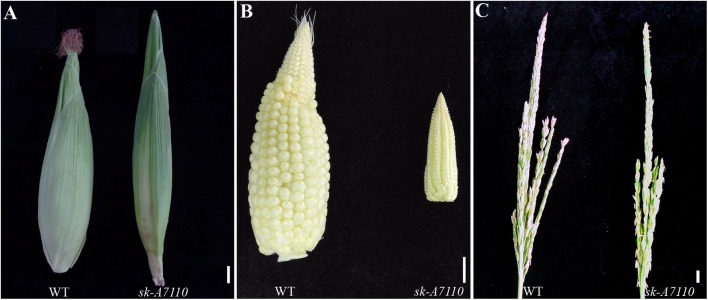
Phenotypic comparison of wild type and *sk-A7110* plants. **(A)** Wild type (WT) and *sk-A7110* ears. The *sk-A7110* completely lacked silks. **(B)** WT and *sk-A7110* ears without husk leaves. Ear volume of *sk-A7110* is smaller than WT. **(C)** WT and *sk-A7110* tassels. The number of tassel branches of *sk-A7110* was less than that of the WT. Bars = 1 cm.

In 2012, we identified the *sk-A7110* mutant in the field. Phenotypic observation showed that there was no difference in vegetative growth, plant height, ear height, pollen viability, or other traits between this mutant and the WT (results not shown), but the mutant completely lacked silks (**Figure 1A**). In addition, the volume of the ear was substantially smaller in the mutant than in WT, although the cob retained the typical conical shape (**Figure 1B**). Moreover, some female spikelet of the mutant had male characteristics, such as, some ears had male spikelet at the top (**Supplementary Figure S1A**), and some ears had yellow anthers (**Supplementary Figure S1B**).

We also detected a significant decrease in tassel branching in *sk-A7110* compared to WT (**Figure 1C**). Therefore, we counted the numbers of tassel branches in WT and mutant plants in three segregating populations. The number of branches, especially secondary branches, was significantly reduced in *sk-A7110* tassels compared to WT (**Table 1**), indicating that tassel branch differentiation is inhibited in this mutant.

**Table 1:** Number of branches per tassel of *sk-A7110* and WT.

Combinations	Branch type	Average branch no. of wild type (N = 30)	Average branch no. of *sk-A7110* (N = 30)
80044 /*sk-A7110//sk-A7110*	Primary branch	8.89	6.24^∗∗^
	Secondary branch	2.11	0.83^∗∗^
B73 /*sk-A7110*//*sk-A7110*	Primary branch	11.65	9.58^∗∗^
	Secondary branch	3.72	1.65^∗∗^
*+/sk-A7110*/*sk-A7110*	Primary branch	7.95	3.95^∗∗^
	Secondary branch	2.45	0.3^∗∗^


*^∗∗^Indicates the different is significance (Student’s *t*-test: ^∗∗^*P* < 0.01).*

### The Pistil Primordium of *sk-A7110* Degenerates During the Late Stage of Floret Differentiation

**FIGURE 2:**
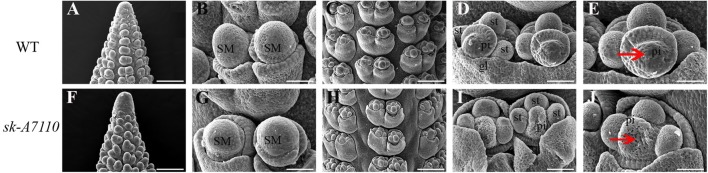
Development of young ears in WT and *sk-A7110.* **(A,F)** Ear at the elongating stage. **(B,G)** Ear at the spikelet late-differentiation stage. **(C**,**H)** Ear at the floret pre-differentiation stage. Every floret includes a pair of glumes, three stamen primordia, and one pistil primordium. **(D,E)** Normal ear at the floret late-differentiation stage. The pistil primordia bulged and began to elongate rapidly. **(I,J)** The *sk-A7110* ear at the floret late-differentiation stage. The pistil primordia shrinked and began to degenerate (Red arrowhead).SM, Spikelet meristem; st, Stamen primordium; pi, Pistil primordium; gl, Glume. Bars = 100 μm in **(A**,**C**,**F**,**H)** and 50 μm in **(B,D,E,G,I,J)**.

**FIGURE 3:**
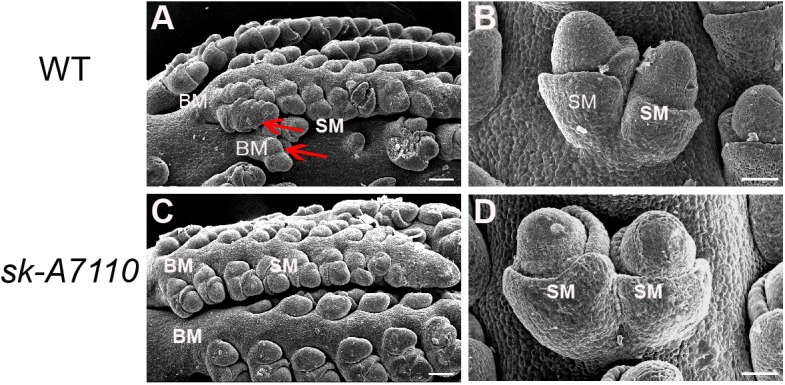
Development of young tassels in WT and *sk-A7110*. **(A,C)** WT and *sk-A7110* tassel in the late stage of spikelet differentiation. The WT had more branching meristems (Red arrowhead). **(B,D)** SMs in WT and *sk-A7110* tassel. BM, Branching Meristem; SM, Spikelet Meristem. Bars = 50 μm.

To identify the origin of the silkless and phenotype, we performed scanning electron microscopy of female panicles at different developmental stages in WT and *sk-A7110* plants. In the WT ears, the pistil primordia bulged and elongated rapidly, beginning at the last stage of floret differentiation (**Figures 2D,E**). However, in *sk-A7110* ear, the pistil primordia shrinked and began to degenerate at the same stage (**Figures 2I,J**), which is completely opposite to WT. By contrast, other developmental stages of the ear, such as apical cone elongation (**Figures 2A,F**), spikelet differentiation (**Figures 2B,G**), and floret differentiation (**Figures 2C,H**) were the same in the mutant and the WT. These results suggest that during ear development, the defects in *sk-A7110* first occur during the last stage of floret differentiation. We also observed the differentiation of tassel by scanning electron microscope. It was found that the *sk-A7110* mutant had fewer branching meristems than the WT (**Figures 3A,C**), but there is no distinct difference in the spikelet meristem (**Figures 3B,D**).

### Genetic Analysis and Mapping of *sk-A7110* Gene

All F_1_ plants were normal, and the phenotypes segregated in the three BC_1_ population was consistent with a WT: mutant ratio of 1:1, as determined by Chi-square testing (**Table 2**). Taken together, these results indicate that *sk-A7110* is controlled by a single recessive nuclear gene.

**Table 2:** The genetic analysis of *sk-A7110* mutant.

Populations	Total No	No. of wild type	No. of mutant	Theoretical ratio	( X^2^ 0.05 ≤ 3.84)
*80044 /sk-A7110// sk-A7110*	938	465	473	1:1	0.68
*B73 /sk-A7110// sk-A7110*	231	117	114	1:1	0.4
*P2 /sk-A7110// sk-A7110*	2785	1422	1363	1:1	1.25


**FIGURE 4:**
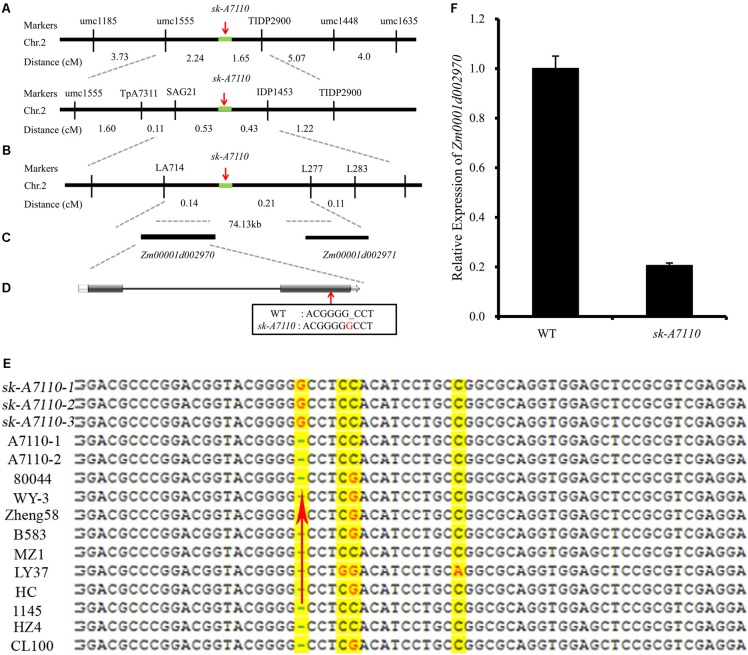
Map-based cloning of the *sk-A7110* locus. **(A)** The *sk-A7110* locus was primary delimited to the interval between the *umc1555* and *umc1448* on chromosome 2. **(B)** The *sk-A7110* locus was finally delimited to an interval about 74.13 kb flanked by *LA714* and *L277*. **(C)** Two genes predicated in the 74.13 kb interval. **(D)** Structure of the candidate gene for *sk-A7110*, the red arrow shows the mutation site. **(E)** Sequence comparison of *Zm00001d002970* between the *sk-A7110* mutant and 10 maize inbred lines. The red arrow shows the mutation site. **(F)** QRT-PCR analysis of *Zm00001d002970* in WT and *sk-A7110*. Error bars indicating SD obtained from three biological repeated.

We mapped the *sk-A7110* gene using bulked segregant analysis (BSA) and simple sequence repeat (SSR) markers. Initially, we screened 228 SSR markers distributed over all 10 chromosomes and identified 37 polymorphic markers between P2 and the mutant. These 37 markers were used to screen DNA pools and identified multiple markers on chromosome 2 that might co-segregate with the *sk-A7110* gene. Subsequently, analysis of 134 mutant individuals derived from a P2/*sk-A7110*//*sk-A7110* population showed that two SSR markers (*umc1555* and *umc1448*) on the long arm of chromosome 2 are linked to *sk-A7110*, at a genetic distance of 2.24 and 6.72 cM, respectively (**Figure 4A**).

We then designed 292 pairs of specific primers between *umc1555* and *umc1448* and used 2785 individuals with mutant phenotypes from the P2/*sk-A7110*//*sk-A7110* population and 938 mutant individuals from the 80044/*sk-A7110*/*sk-A7110* population for fine mapping. This ultimately delimited the *sk-A7110* locus to a 74.13-kb region between *LA714* and *L277* (**Figure 4B**).

### Candidate Gene Annotation and Sequence Analysis

Two gene models were predicted in the 74.13-kb target region based on the maize B73 sequence (version 4.0)^2^ (**Figure 4C**). Gene annotation showed that one of these genes, *Zm00001d002970*, encodes an uncharacterized family 1 uridine diphosphate glycosyltransferase and the other, *Zm00001d002971*, encodes a DUF177 domain protein. DNA sequence comparison between *sk-A7110* and WT revealed that a single nucleotide, G, was inserted at base 569 downstream of the ATG start codon of *Zm00001d002970* in *sk-A7110* (**Figure 4D**); this resulted in the encoded amino acid is inconsistent from the 190th, and the number of encoded amino acids is reduced from 511 to 297. No sequence difference was found in *Zm00001d002971* between *sk-A7110* and WT. Sequencing primers for candidate genes are shown in **Supplementary Table S2**.

To verify the specificity of the mutation, we analyzed the full-length genomic sequences of *Zm00001d002970* in inbred lines 80044, WY-3, Zheng58, B583, MZ1, LY37, HC, 1145, HZ4 and CL100 and found that G was inserted in this gene only in the mutant (**Figure 4E**). Moreover, qRT-PCR analysis revealed that *Zm00001d002970* was expressed at significantly lower levels in *sk-A7110* than in WT, indicating that its expression is significantly inhibited in the mutant (**Figure 4F**). Based on these results, we identified *Zm00001d002970*, which encodes UDP-glycosyltransferase, as the candidate gene responsible for the *sk-A7110* phenotype.

### Transcriptome Analysis

To examine the genes and pathways affected by the mutation of *SK-A7110*, we conducted mRNA sequencing (RNA-seq) of the WT and *sk-A7110* to identify differentially expressed genes (DEGs) in ear and tassle inflorescence. This identified 258 DEGs based on the criteria of corrected *p-value <* 0.05 between WT ears and *sk-A7110* ears, including 119 up- and 139 down-regulated DEGs (**Supplementary Data Sheet S1**). Similarly, 43 DEGs were found in WT and *sk-A7110* tassels (**Supplementary Data Sheet S2**), among which 33 were up-regulated and 11 were down regulated. To validate the RNA-seq expression data, we subjected 15 randomly selected DEGs to qRT-PCR. The qRT-PCR results were in good agreement with the RNA-seq results, indicating that the RNA-seq data were highly reliable (**Table 3**).

**Table 3:** The expression value of 15 selected genes in RNA-seq and real time PCR.

Genes	RNA-seq		qRT-PCR	
		
	WT	*sk-A7110*	WT	*sk-A7110*
*Zm00001d011687*	24.806	690.594	1	22.471
*Zm00001d004417*	141.777	0.333	1	0.029
*bde*	1036.838	493.030	1	0.853
*lox10*	650.960	4962.510	1	2.072
*ra1*	162.020	85.307	1	0.406
*ZAG1*	483.691	120.439	1	0.246
*ZAG2*	230.209	10.716	1	0.024
*zmm2*	5.412	1.012	1	0.692
*zmm6*	1552.138	439.914	1	0.161
*UB2*	16.087	6.210	1	0.411
*UB3*	9.367	3.830	1	0.378
*Zm00001d033020*	14.217	3.710	1	0.316
*Zm00001d052543*	5.927	13.823	1	2.870
*Zm00001d042922*	5.960	1.803	1	0.445
*Zm00001d049950*	2.387	5.987	1	1.878


**FIGURE 5:**
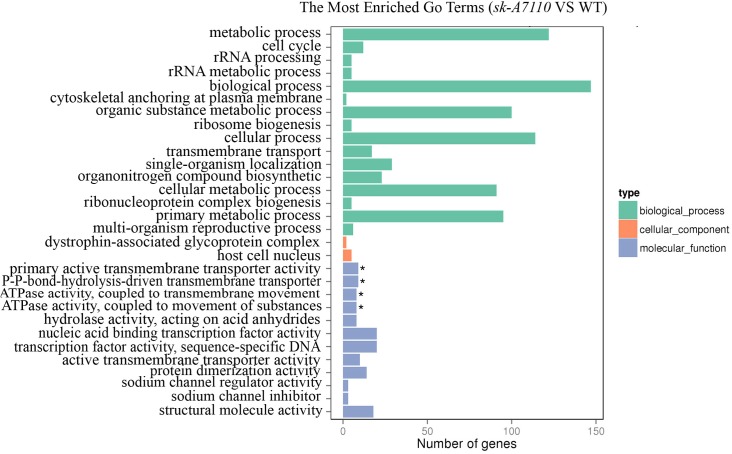
The enriched GO terms in WT and *sk-A7110.* Differentially expressed genes were summarized in the three main GO categories: biological process (Green box), cellular components (Orange red box) and molecular functions (Blue box). The *x*-axis indicates the gene number, and the *y*-axis indicates the subcategories. Black asterisks indicate significantly enriched terms. The top 30 enriched GO terms are listed.

To further explore the functions of the DEGs, we performed GO annotation and GO enrichment analysis (corrected *p-value <* 0.05). For DEGs between the WT and mutant ears, the significantly enriched GO terms included the following: primary active transmembrane transporter activity (GO:0015399), P-P-bond-hydrolysis-driven transmembrane transporter activity (GO:0015405), ATPase activity, coupled to transmembrane movement of substances (GO:0042626) and ATPase activity, coupled to movement of substances (GO:0043492) (**Figure 5**). All of these terms belong to the molecular function group, suggesting that *SK-A7110* may play significant roles in transmembrane transport of substances.

Among the DEGs that were down-regulated in the mutant ears, the most significantly enriched GO terms were nucleic acid binding transcription factor activity (GO:0001071) and transcription factor activity, sequence-specific DNA binding (GO:0003700). A total of 28 differentially expressed transcription factor genes, in multiple families, were detected in WT vs. *sk-A7110* (**Supplementary Table S4**); these included MADS-box, AP2-EREBP, MYB, and bHLH transcription factors. These findings indicate that these transcription factors play important roles in sex determination in maize inflorescences and provide clues for further exploration of inflorescence development in maize.

KEGG analysis identified 139 DEGs in ears with KEGG annotations, and these DEGs are enriched in 66 pathways mainly involved in biochemical metabolism and signal transduction. No significantly enriched pathway was found according to the KEGG screening standard (corrected *p-value <* 0.05), which may be due to the sampling period was a little late. However, the top pathway of our KEGG analysis results is mainly related to the metabolism of a variety of amino acids, ascorbic acid metabolism, and α-linolenic acid metabolism (JA biosynthesis) (**Supplementary Table S5**).

**FIGURE 6:**
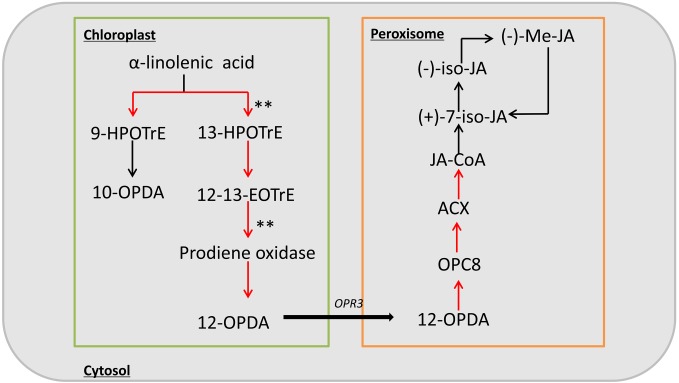
Schematic diagram of the jasmonic acid biosynthesis pathway. Red arrow indicates up-regulation of genes during this process in *sk-A7110* compared with WT; ^∗∗^indicate the position of significant differentially expressed genes.

Previous studies have shown that the inflorescence development is closely related to endogenous hormones in plants. Therefore, we checked more in detail hormone related pathways, and found that only JA synthesis pathway is relatively enriched. In addition, studies of the *ts1* and *ts2* mutants have shown that JA is involved in apoptosis in maize pistils, so we focused on the JA biosynthesis pathway. Based on the RNA-seq data, among the 31 genes in this pathway, 17 genes were upregulated in the mutant, including nine with a | log_2_ ratio|≥ 1 (**Supplementary Table S6**). These DEGs included two significantly up-regulated genes encoding key enzymes of jasmonic acid biosynthesis, lipoxygenase (LOX10) and allene oxide synthase (AOS) (**Figure 6**). These results suggest that the phenotype of *sk-A7110* is likely associated with effects on JA metabolism.

Analysis of the RNA-seq data of tassels found that DEGs were mainly enriched in amino acid metabolism and secondary biomass metabolic pathways. And in the down-regulated expression gene, two genes associated with the tassel branch of maize, *UB2* and *UB3*, were down-regulated significantly (**Table 4**). Both *UB2* and *UB3* are member of the SQUAMOSA promoter binding protein-like (*SPL*) gene family, and their expression levels were down-regulated in both *ub2* and *ub3* mutants with the number of tassel branches being significantly reduced (Chuck et al., 2014; Du et al., 2017). This indicates that *sk-A7110* affects the number of tassel branches in maize by regulating the expression of *UB3* and *UB2*. Further analysis of the RNA-seq data of ears revealed that three genes involved in maize inflorescence development, *ZAG1*, *ZAG2* and *bearded-ear 1 (bde1)*, are significantly down-regulated in *sk-A7110* (**Table 4**). Together, these results suggest that *SK-A7110* regulates maize inflorescence development in conjunction with multiple inflorescence development-related genes.

**Table 4:** RNA-seq results for known genes related to maize inflorescence development.

Gene ID	Gene name	log2 Fold Change	Gene description	Reference
*Zm00001d037737*	*ZAG1*	-2.0058	MADS transcription factor	Mena et al., 1996
*Zm00001d041781*	*ZAG2*	-4.4251	MADS transcription factor	Schmidt et al., 1993
*Zm00001d017614*	*bde1*	-1.0724	bearded-ear1	Thompson et al., 2009
*Zm00001d031451*	*UB2*	-1.3470	unbranched2	Chuck et al., 2014
*Zm00001d052890*	*UB3*	-1.3170	Unbranched3	Du et al., 2017


### Endogenous Hormone Concentrations

**FIGURE 7:**
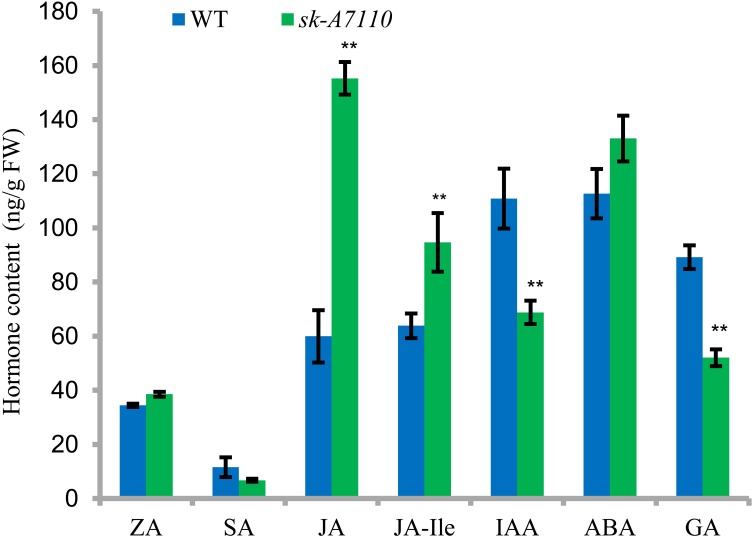
Endogenous hormone content in WT and *sk-A7110* young ears. ZA, zeatin; SA, salicylic acid; JA, jasmonic acid; JA-Ile, JA-Isoleucine; ABA, abscisic acid; GA, gibberellic acid. ^∗∗^Indicates significantly different (*P* < 0.01, Student’s *t*-test). FW, fresh weight. Error bars indicating SD obtained from three biological repeated.

We measured endogenous hormone levels in young ears of WT and mutant plants. Levels of salicylic acid (SA), GA, and the auxin indole acetic acid (IAA) were lower in *sk-A7110* than in WT, whereas JA, ABA, and JA-Ile levels were higher in *sk-A7110* than in WT and there was little difference in zeaxanthin (ZA) levels (**Figure 7**). Among these phytohormones, the contents of JA, JA-Ile, IAA and GAs significantly differed between the two lines. These results indicate that the mutant phenotype is closely related to endogenous hormone levels.

### Allelism Test of the *sk-A7110* and *sk1* Mutants

We made a cross with a *sk-A7110/SK-A7110* heterozygous plant as the female parent and *sk1*/*sk1* as the male parent. The resulting progeny included silkless plants, with a 1:1 ratio of normal to mutant plants, indicating that *sk-A7110* and *sk1* are allelic and therefore are caused by mutations in the same gene. Indeed, previous positional cloning and transgenic analysis found that the *SK1* gene encodes a UDP-glycosyltransferase (Hayward et al., 2016). The target genes of *SK1* and *Zm00001d002970* are identical, again confirming that *Zm00001d002970* is the gene responsible for the *sk-A7110* phenotype.

## Discussion

During the early 20th century, many studies focused on ear and silk mutants in maize. The *ts1* (Schmidt et al., 1993) and *ts2* (DeLong et al., 1993) mutants are characterized by feminization of the male and female spikelets with multiple filaments. In the *bd1* mutant, the ears are branched and lack florets and filaments; *BD1* encodes an ERF transcription factor that regulates differentiation of the spikelet meristems into florets (Chuck et al., 2002). The female spikelets of *silky-I* (*si1*) have four filaments and the excess filaments are stamens in the upper florets that failed to abort (Fraser, 1933; Ambrose et al., 2000). The *bearded-ear* (*bde*) mutant has several small flowers in its spikelet and the ovules contain extra silks with lower fertility (Kempton, 1934; Thompson and Hake, 2009). *Silkless1 (sk1)*, an important masculinized mutant, has tassel florets like those of WT but with aborted pistils in female spikelets, leading to the complete loss of silks (Jones, 1925). Among abnormal inflorescence maize mutants described to date, only *sk1* lacks silks but exhibits no other mutant phenotype, indicating that *SK1* plays a specific role in pistil development. Jones subsequently discovered several other filament-deficient mutants with anthers at the tops of ears, which together with *sk1* are referred to as “*sk*” mutants (Jones, 1934). In the current study, we discovered the female-sterile mutant *sk-A7110*, a “*sk*”-type mutant (**Figure 1A**). Scanning electron microscopy showed that the pistils of *sk-A7110* degenerate beginning at the late floret differentiation stage (**Figures 2D,E,I,J**), suggesting that *SK-A7110* is essential for late-stage pistil development in maize.

Map-based cloning and allelism test revealed that *sk-A7110* and *sk1* are allelic mutants with mutations in a gene encoding a UDP-glycosyltransferase. The *sk1-Allie1* mutant contains a novel 3549-bp insertion in the intron of *SK1* (Hayward et al., 2016) and the *sk-A7110* mutant contains an insertion of G at position 569 downstream of the translational start site, resulting in a frame shift and early translational termination (**Figure 4D**). Different allelic mutants can have some perceptible differences in phenotype due to their different mutation sites. For example, the *sk1* mutant showed a complete absence of silks, whereas *sk-A7110* not only showed a loss of silks, but also significantly reduced tassel branching (especially secondary branches) (**Table 1**). Therefore, discovering and analyzing different *SK1* alleles can provide new information about the function of *SK1* and the regulatory mechanism of pistil abortion in the maize inflorescence.

RNA-seq analysis showed that multiple genes associated with maize inflorescence development were differentially expressed in *sk-A7110* vs. WT. Most of these DEGs are MADS-box family genes, AP2-EREBP, MYB, and bHLH transcription factors, and *ZAG1*, *ZAG2*, and *BDE* were the most distinct genes (**Table 4**). Many studies have proved that MADS-box and AP2-EREBP transcription factors play important roles in floral organ morphogenesis in maize and other plants (Irish and Sussex, 1990; Bowman et al., 1991; Hollick et al., 2005; Parkinson et al., 2007; Banks, 2008; Gallavotti et al., 2010; Zhang et al., 2012; Tanaka et al., 2013; Lu et al., 2017). MADS-box mutants, *zag1* (Mena et al., 1996), *zag2* (Schmidt et al., 1993) and *bde1* (Thompson et al., 2009) exhibit abnormal inflorescence development, such as extra carpels emerging on female ears and pistils in the tassel that fail to abort. Reverse genetics studies have also suggested that MYB transcription factors play major roles in regulating floral organ development through a JA-mediated signaling pathway. For example, the *Arabidopsis myb21* mutant is characterized by shorter filaments, less dehiscence of pollen sacs and lower viability of pollen grains compared to WT (Cheng et al., 2009). In addition, *UB2* and *UB3*, which related to maize tassel branching were also significantly down-regulated in *sk-A7110*, and it is maybe the main reason for reduced tassel branching. So, our results demonstrate that *SK-A7110* plays an important role in regulating maize inflorescence development in coordination with multiple genes.

In the current study, RNA-seq analysis revealed that most genes involved in the JA pathway are upregulated in *sk-A7110* (**Figure 6** and **Supplementary Table S5**). The contents of JA and JA-Ile were significantly higher in *sk-A7110* ears than that in WT (**Figure 7**), which is consistent with the RNA-seq results. Meanwhile, analysis of *sk1* indicated that *SK1* affects JA accumulation and that ectopic expression of *SK1* protects the pistils in tassels, thus leading to complete feminization, while the contents of JA and its direct precursor were significantly reduced in feminized tassels (Hayward et al., 2016). These results indicate that *SK-A7110* plays an important role in JA metabolism during inflorescence development in maize and that JA and associated substances play an important role in determining the fate of maize pistils, with high JA levels promoting pistil abortion.

In addition, although the result of hormone measurement showed that the content of IAA and GA in ear of *sk-A7110* was significantly lower than that in WT, we believe they were only the result caused by the undeveloped silks, not the cause of the mutant phenotype. Because it was found that in maize the development silks can produce high levels of GA (Dellaporta and Calderon-Urrea, 1994; Eveland et al., 2014), so when silks are not developed, the GA content in the ear decreased significantly compared to WT. And, filaments stop developing, with corresponding reduction in the content of IAA, which is closely related to growth and development. In addition, exogenous GA and IAA did not restore the mutant phenotype (results not shown). Therefore, combining the two results we make the speculation that the significant increase in JA content in the ear is the reason for the absence of silks in *sk-A7110*, while the decrease in IAA and GA content are the result of the absence of silks.

Glycosylation, which is catalyzed by glycosyltransferases, is an important way in which plants regulate hormone activity levels. For example, glycosylation of plant hormones is a major way in which plant hormones are inactivated, ultimately regulating plant growth and development through synergistic effects with glycosylation products (Wang, 2009). To date, a number of hormone-related glycosyltransferase genes have been cloned and identified, such as the IAA glycosyltransferase gene *UGT73E2* (Tognetti et al., 2010), the cytokinin glycosyltransferase *UGT76C1* (Wang et al., 2013), the ABA glycosyltransferase gene *UGT71B6* (Priest et al., 2006), the brassinosteroid glycosyltransferase gene *UGT73C6* (Husar et al., 2011) and the SA glycosyltransferase gene *OsSGT1* (Umemura et al., 2009). Although a JA-independent glycosyltransferase has not yet been reported, JA was shown to interact with auxin to regulate plant growth and development (Grunewald et al., 2009). Song (2005) reported that the *Arabidopsis* auxin glycosyltransferase gene *AtJGT1* glycosylates modified JA *in vitro* (Song, 2005), but whether this protein affects the dynamic balance of JA in plants remains unclear.

In our study, map-based cloning indicated that *sk-A7110* encodes a UDP-glycosyltransferase, and GO-enrichment analysis showed that the significantly enriched GO terms of the DEGs are related to the transmembrane transport of substances. In addition, analysis of *sk1* indicated that SK1 localizes to the peroxisome (Hayward et al., 2016). Therefore, we hypothesize that the glycosyltransferase encoded by *SK-A7110* glycosylates JA and that it is active during its transmembrane transport from the peroxisome, thus maintaining the dynamic balance of JA within the pistil.

## Author Contributions

BL designed the experiments. YaZ performed most of the experiments and analyzed the data. Other authors assisted in experiments and discussed the results. YaZ and YoZ wrote the manuscript.

## Conflict of Interest Statement

The authors declare that the research was conducted in the absence of any commercial or financial relationships that could be construed as a potential conflict of interest.

## Footnotes

1 http://maizecoop.cropsci.uiuc.edu/

2 http://www.maizesequence.org

## Supplementary Material

FIGURE S1 Other characteristics of some ears. **(A)** Some male spikelet at the top of the ear (red arrow). **(B)** Yellow anthers in the ear (red arrow).

TABLE S1 Polymorphic molecular markers used for gene mapping.

TABLE S2 The primers information of candidate genes for *sk-A7110* sequencing.

TABLE S3 The primers used for qRT-PCR. ACTIN refers to the Actin-1 gene (*Zm00001d010159*).

TABLE S4 Transcription factors differentially expressed in *sk-A7110* and wild type ears.

TABLE S5 The first 10 paths of KEGG analysis of the RNA-seq data of ears.

TABLE S6 RNA-seq analysis of genes related to JA synthesis.

DATA SHEET S1 The DEGs data of *sk-A7110* ears vs. WT ears.

DATA SHEET S2 The DEGs data of *sk-A7110* tassels vs. WT tassels.
