# Supplementary figures and images for: Mapping and Functional Analysis of a Maize Silkless Mutant sk-A7110
Source: Front Plant Sci. 2018 Aug 21;9:1227. doi: 10.3389/fpls.2018.01227 (PMC6111845; doi:10.3389/fpls.2018.01227)

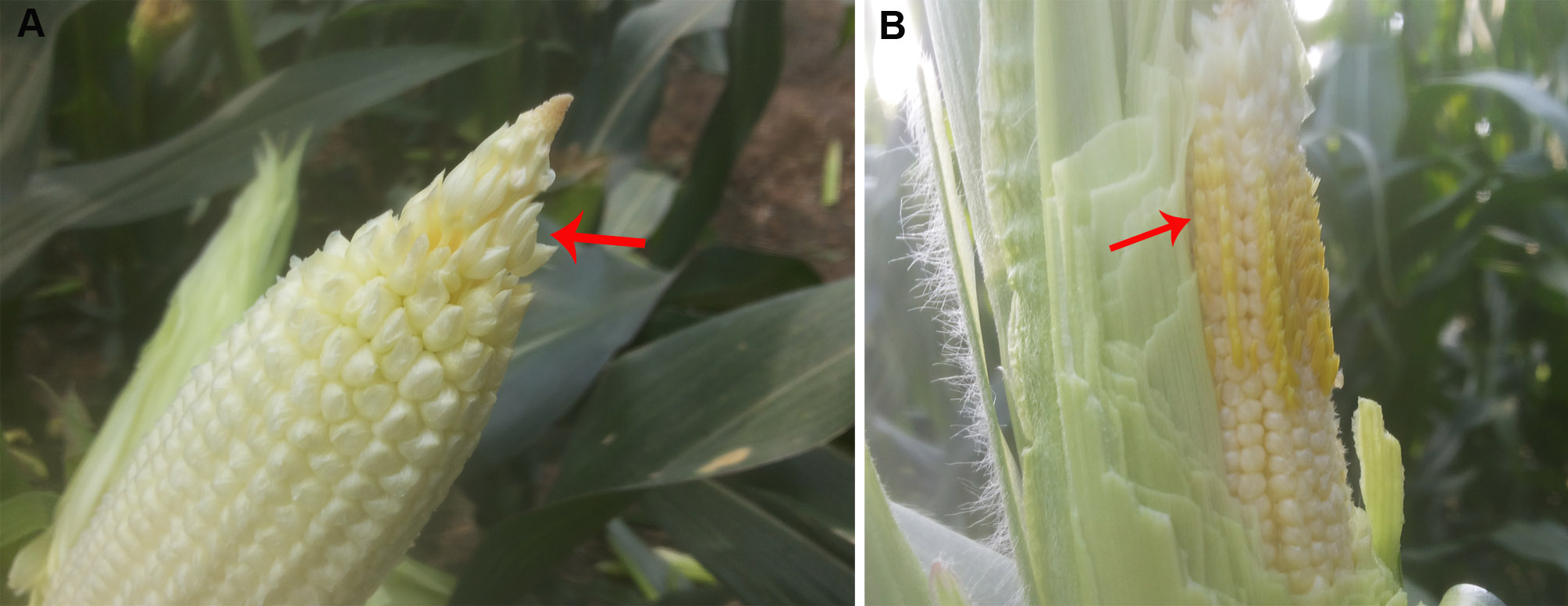

Supplement: FIGURE S1 — Other characteristics of some ears. (A) Some male spikelet at the top of the ear (red arrow). (B) Yellow anthers in the ear (red arrow). [file Image_1.JPEG]
