# Supplementary material for: Mapping and Functional Analysis of a Maize Silkless Mutant sk-A7110
Source: Front Plant Sci. 2018 Aug 21;9:1227. doi: 10.3389/fpls.2018.01227 (PMC6111845; doi:10.3389/fpls.2018.01227)
Supplement: TABLE S1 — Polymorphic molecular markers used for gene mapping. [file Table_1.DOCX]

**Supplementary Table 1 Polymorphic molecular markers used for gene mapping**

| Makers | Forward primer (5'–3') | | Reverse primer (5'–3') |
| --- | --- | --- | --- |
| p-umc1555 | | ATAAAACGAACGACTCTCTCACCG | ATATGTCTGACGAGCTTCGACACC |
| p-umc1448 | ATCCTCTCATCTTTAGGTCCACCG | | CATATACAGTCTCTTCTGGCTGCTCA |
| p-umc1185 | AGTAAAAGAGGCAAGGACTACGGC | | GCGGCGATATATACGAGGTTGT |
| p-umc1635 | GCTGAGCAGATCTTTCCTTGTTTC | | AAGGAGCAGAACTCGGAGACG |
| TIDP290 | TGCCTACCATACAAATCCTCG | | CGTGCTACCTAGTGAAGGGC |
| LAG21 | TTCATAAGTAATCCGGGTAG | | CATAGTATCCACGTCCATCC |
| IDP1453 | TGCAATCTCAACATTCAGGG | | CGACTCGGTCATCATAGTGC |
| L277 | CTGGCGTATGACGAGAACC | | ATTGCCTTTGAGCCTTTCC |
| LA7-14 | TCGCCTCCGAGACACTTG | | TGGCCTGGCCTCTACCTAT |
