# Supplementary material for: Mapping and Functional Analysis of a Maize Silkless Mutant sk-A7110
Source: Front Plant Sci. 2018 Aug 21;9:1227. doi: 10.3389/fpls.2018.01227 (PMC6111845; doi:10.3389/fpls.2018.01227)
Supplement: TABLE S2 — The primers information of candidate genes for sk-A7110 sequencing. [file Table_2.DOCX]

**Supplementary Table 2 The primers information of candidate genes for *sk-A7110* sequencing**

| Gene | Primers | Forward primer (5'–3') | Reverse primer (5'–3') |
| --- | --- | --- | --- |
| *Zm00001d002971* | UP-1 | CCCTCCATCCCAAAAAACAA | CACGGGCACGCTCATCATA |
|  | UP-2 | TGTCTCGATCCAGAGCCTCC | AGTTACAAACCACCAACCTTCC |
|  | UP-3 | GAAGAAGACTTCCAGCCAAAGG | CAGACTCGTACCCAAAACCATG |
| *Zm00001d002970* | UDP-1 | AATCTTAGGCGTTGTTTGGT | CTGAGCTGCTTCACTCTTTCT |
|  | UDP-2 | GGCATCGATATCGTCCAGC | GGCATAGCAACTTCCACCTG |
|  | UDP-3 | CGTAAAGCAGTCACAACTCACA | GCAGGAAAAAAAAAGAAAAGAA |
|  | UDP-4 | CCATCCTCGCCATTTCTGAGAT | AAGCAGCCAGCAGTTTTTTTTT |
|  | UDP-5 | CTGACGATATGATGTGACGAGTG | GATGTAGGCGCAGTTGATGAAC |
