# Supplementary material for: Mapping and Functional Analysis of a Maize Silkless Mutant sk-A7110
Source: Front Plant Sci. 2018 Aug 21;9:1227. doi: 10.3389/fpls.2018.01227 (PMC6111845; doi:10.3389/fpls.2018.01227)
Supplement: TABLE S3 — The primers used for qRT-PCR. ACTIN refers to the Actin-1 gene (Zm00001d010159). [file Table_3.DOCX]

**Supplementary Table 3 The primers used for qRT-PCR**

| Genes | Forward primer (5'–3') | Reverse primer (5'–3') |
| --- | --- | --- |
| *ZAG1* | GAAGCTGAAGGAGCCCATATC | TTGATCTCAGTCTTGCCCTTG |
| *ZAG2* | TAAGATCAGGGCCAGGAAGA | CTGAGGGTCATGTGGTCATTC |
| *BDE* | GCATCCAACAACTGCTCAATC | GGGCTCCTTGGGATATTATTGG |
| *ZMM6* | CCTGAAACAGCACTCCAGAATA | GTTCCTTTGAGTCCGCTGTAA |
| *Zm00001d053675* | CTTCGGCCAGTACCACTTC | GCTTGAGGAACTTCTCCATCTC |
| *ZMM2* | GTTATAGATCGGTACGGCAAGG | GTTGTGCAGTTGTTGTCTCAAG |
| *Zm00001d011687* | CTGCAGGGCACAGTAGTC | AATATGGCCTCGACGTTCTC |
| *Zm00001d004417* | TGGATTCAGATCCCGGAGAA | GTTAGGGAGAATGCCCTTACG |
| *Ra1* | AGGAGTTCAGATCAGCACAAG | AGCAGCAATACGGTGTGAA |
| *Zm00001d002970* | CCACTGCGGATGGAACTC | GATGTAGGCGCAGTTGATGA |
| *Zm00001d049950* | CGCAGCAACCACCAACTTCTA | CGCCAGCGACTTCTTCACCT |
| *Zm00001d033020* | AAACTCCGCCGCCCTCAA | TCCGCCATGCACCACTCG |
| *Zm00001d052543* | CTTGGAGGAGATCCGGGTG | CGTCGTGGTGGGTGAGGG |
| *Zm00001d042922* | ACAAGGGTGAGGAGAAGCAG | TCAAGACCGTAGGCAATAGC |
| *UB2* | GGCAGGCTCAGAAGCTTCA | TACCACCCTCGCTCCCATA |
| *UB3* | TCCTCCTCCCCCTCACCAT | ACTCCTGCGAGACATCCACC |
| *ACTIN* | TCACCCTGTGCTGCTGACCG | GAACCGTGTGGCTCACACCA |

Note: *ACTIN* refers to the *Actin-1* gene (*Zm00001d010159*)
