# Supplementary material for: Mapping and Functional Analysis of a Maize Silkless Mutant sk-A7110
Source: Front Plant Sci. 2018 Aug 21;9:1227. doi: 10.3389/fpls.2018.01227 (PMC6111845; doi:10.3389/fpls.2018.01227)
Supplement: TABLE S4 — Transcription factors differentially expressed in sk-A7110 and wild type ears. [file Table_4.DOCX]

**Supplementary Table 4 Transcription factors differentially expressed in *sk-A7110* and wild type ears**

| Gene ID | Type | Gene name | log2FoldChange | Gene description |
| --- | --- | --- | --- | --- |
| *Zm00001d048082* | MADS | *zmm14* | -1.2865 | MADS14%2C transcript variant X2 |
| *Zm00001d031620* | MADS | *zmm6* | -1.819 | MADS6 |
| *Zm00001d037737* | MADS | *ZAG1* | -2.0058 | homologue of Arabidopsis gene AGAMOUS |
| *Zm00001d017614* | MADS | *bde1* | -1.0724 | bearded-ear1 |
| *Zm00001d041781* | MADS | zag2 | -4.4251 | AGAMOUS homolog2%2C transcript variant X2 |
| *Zm00001d006094* | MADS | *zmm27* | -1.2736 | MADS27 |
| *Zm00001d021057* | MADS | *MADS4* | -1.6517 | MADS-domain transcription factor 4 |
| *Zm00001d028217* | MADS | *LOC103632725* | -1.7923 | MADS-box transcription factor 1-like%2C transcript variant X2 |
| *Zm00001d018416* | bHLH | *bHLH168* | -0.9339 | putative HLH DNA-binding domain superfamily protein%2C transcript variant X2 |
| *Zm00001d000236* | bHLH | *LOC103646088* | 1.0706 | anthocyanin regulatory R-S protein-like%2C transcript variant X3 |
| *Zm00001d018825* | bZIP | *LOC103631951* | 0.86265 | uncharacterized LOC103631951 |
| *Zm00001d038584* | AP2-EREBP | *LOC103630552* | -0.90745 | ethylene-responsive transcription factor 12-like |
| *Zm00001d043204* | AP2-EREBP | *LOC103651111* | -1.3451 | ethylene-responsive transcription factor 12-like |
| *Zm00001d010676* | AP2-EREBP | *EREB93* | -0.64652 | putative AP2/EREBP transcription factor superfamily protein |
| *Zm00001d005951* | HB | *si606017c07* | 0.67952 | LOC100194336 |
| *Zm00001d032194* | MYB | *LOC100273887* | -1.0127 | uncharacterized LOC100273887 |
| *Zm00001d039371* | TCP | *umc2049* | 0.68043 | LOC100281134%2C transcript variant X2 |
| *Zm00001d028216* | C2C2-YABBY | *ZmDL1* | -1.9635 | protein DROOPING LEAF |
| *Zm00001d002185* | SET | *LOC100283168* | -0.83809 | MYND finger family protein |
| *Zm00001d024376* | WRKY | *WRKY67* | -1.2655 | WRKY25 - superfamily of TFs having WRKY and zinc finger domains |
| *Zm00001d014858* | HMG | *HMG13* | 0.57114 | high mobility group protein1 |
| *Zm00001d048552* | SNF2 | *LOC103639629* | -0.94978 | chromodomain-helicase-DNA-binding protein 1-like%2C transcript variant X2 |
| *Zm00001d033048* | Tify | *LOC103643602* | 3.4542 | protein TIFY 10A-like |
| *Zm00001d003822* | S1Fa-like | *LOC103646512* | -0.96108 | DNA-binding protein S1FA |
| *Zm00001d004881* | OFP | *LOC103647057* | 1.2581 | uncharacterized LOC103647057 |
