# Supplementary material for: Mapping and Functional Analysis of a Maize Silkless Mutant sk-A7110
Source: Front Plant Sci. 2018 Aug 21;9:1227. doi: 10.3389/fpls.2018.01227 (PMC6111845; doi:10.3389/fpls.2018.01227)
Supplement: TABLE S5 — The first 10 paths of KEGG analysis of the RNA-seq data of ears. [file Table_5.DOCX]

**Supplement 5 The first 10 paths of KEGG analysis of the RNA-seq data of ears**

| Terms | P-Value | Corrected P-Value | KEGG_ID/KO |
| --- | --- | --- | --- |
| beta-Alanine metabolism | 0.02069 | 0.57512 | zma:100501579\|zma:100274311\|zma:100281700\| |
| Ascorbate and aldarate metabolism | 0.02339 | 0.57512 | zma:100381436\|zma:100381436\|zma:100281700\| |
| Valine, leucine and isoleucine degradation | 0.03095 | 0.57512 | zma:100274311\|zma:100281700\|zma:100283367\| |
| Arginine and proline metabolism | 0.03726 | 0.57512 | zma:100282559\|zma:542400\|zma:100272740\|zma:100281700\| |
| Circadian rhythm - plant | 0.04342 | 0.57512 | zma:100279726\|zma:100279726\|zma:100216856\| |
| Alanine, aspartate and glutamate metabolism | 0.05150 | 0.57512 | zma:100501579\|zma:542400\|zma:100384690\| |
| alpha-Linolenic acid metabolism | 0.10306 | 0.73845 | zma:542150\|zma:542150\| |
| Ubiquitin mediated proteolysis | 0.10466 | 0.73845 | zma:100194284\|zma:100217233\|zma:100216856\|zma:541984\| |
| Mismatch repair | 0.10754 | 0.73845 | zma:100285696\|zma:100279876\| |
| Benzoxazinoid biosynthesis | 0.11916 | 0.73845 | zma:541977\| |
