# Supplementary material for: Mapping and Functional Analysis of a Maize Silkless Mutant sk-A7110
Source: Front Plant Sci. 2018 Aug 21;9:1227. doi: 10.3389/fpls.2018.01227 (PMC6111845; doi:10.3389/fpls.2018.01227)
Supplement: TABLE S6 — RNA-seq analysis of genes related to JA synthesis. [file Table_6.DOCX]

**Supplementary Table 6 RNA-seq analysis of genes related to JA synthesis**

| Genes ID | Readcount-WT | Readcount-*sk-A7110* | Log2 Fold Change | KEGG ID |
| --- | --- | --- | --- | --- |
| *Zm00001d027893 (LOX9)* | 1125.0780 | 1188.1310 | 0.0787 | K00454 |
| *Zm00001d015852 (Lox11)* | 787.1549 | 1609.2100 | 1.0316 | K00454 |
| *Zm00001d025524 (Lox7)* | 8.0538 | 4.4480 | -0.8565 | K00454 |
| *Zm00001d003533 (ts1)* | 312.5286 | 459.9971 | 0.5576 | K00454 |
| *Zm00001d053675 (lox10)* | 650.9598 | 4962.5100 | 2.9304 | K00454 |
| *Zm00001d041204 (lox12)* | 50.7775 | 62.6038 | 0.3021 | K00454 |
| *Zm00001d053586 (aos3)* | 1292.4270 | 1567.836 | 0.2787 | K01723 |
| *Zm00001d048021 (aos1)* | 1130.1010 | 2419.7720 | 1.0984 | K01723 |
| *Zm00001d028282* | 1321.7860 | 2632.5880 | 0.9940 | K01723 |
| *Zm00001d013185* | 188.2704 | 209.3675 | 0.1532 | K01723 |
| *Zm00001d034186* | 52.9215 | 59.6570 | 0.1728 | K01723 |
| *Zm00001d034184* | 0.9712 | 2.0813 | 1.0997 | K01723 |
| *Zm00001d047340* | 1058.8679 | 1140.2953 | 0.1069 | K10525 |
| *Zm00001d029594* | 840.4170 | 910.8326 | 0.1161 | K10525 |
| *Zm00001d011097 (OPR4)* | 9.8446 | 9.7174 | -0.0187 | K05894 |
| *Zm00001d040842 (opr6)* | 195.8590 | 268.4247 | 0.4547 | K05894 |
| *Zm00001d050107 (opr8)* | 1418.7303 | 1342.0367 | -0.0801 | K05894 |
| *Zm00001d032049* | 708.0134 | 525.1527 | -0.4310 | K05894 |
| *Zm00001d027519* | 933.2151 | 858.8303 | -0.1198 | K10526 |
| *Zm00001d045251* | 3058.0314 | 3503.0921 | 0.1960 | K00232 |
| *Zm00001d042884* | 578.6428 | 474.5432 | -0.2861 | K00232 |
| *Zm00001d037521* | 905.1403 | 678.5549 | -0.4157 | K00232 |
| *Zm00001d045606* | 964.3550 | 658.9741 | -0.5493 | K00232 |
| *Zm00001d048890* | 143.1392 | 127.0831 | -0.1717 | K00232 |
| *Zm00001d015992* | 879.3581 | 829.8221 | -0.0836 | K10527 |
| *Zm00001d009182* | 1945.1744 | 1922.3579 | -0.0170 | K10527 |
| *Zm00001d053308* | 1345.6096 | 1226.8826 | -0.1332 | K10527 |
| *Zm00001d015992* | 75.5291 | 43.1101 | -0.8090 | K10527 |
| *Zm00001d018487* | 1797.2770 | 1685.3975 | -0.0927 | K10527 |
| *Zm00001d015128* | 171.6110 | 234.6870 | 0.4516 | K10527 |
| *Zm00001d049882* | 101.4146 | 218.9285 | 1.1102 | K10527 |
